# Immune Parameters and COVID-19 Infection – Associations With Clinical Severity and Disease Prognosis

**DOI:** 10.3389/fcimb.2020.00364

**Published:** 2020-06-30

**Authors:** Milos Jesenak, Miroslava Brndiarova, Ingrid Urbancikova, Zuzana Rennerova, Jarmila Vojtkova, Anna Bobcakova, Robert Ostro, Peter Banovcin

**Affiliations:** ^1^Department of Pediatrics, Jessenius Faculty of Medicine, Comenius University in Bratislava, University Hospital in Martin, Martin, Slovakia; ^2^Department of Pulmonology and Phthisiology, Jessenius Faculty of Medicine, Comenius University in Bratislava, University Hospital in Martin, Martin, Slovakia; ^3^Department of Clinical Immunology and Allergology, University Hospital in Martin, Martin, Slovakia; ^4^Department of Pediatrics, Faculty of Medicine, Children Faculty Hospital, P. J. Safarik University, Kosice, Slovakia; ^5^Department of Pediatric Infectology, Faculty of Medicine, Children Faculty Hospital, P. J. Safarik University, Kosice, Slovakia; ^6^Pneumo-Alergo Centre Ltd., Falck Healthcare Group, Bratislava, Slovakia; ^7^Department of Pediatric Pulmonology and Phthisiology, Faculty of Medicine, National Institute of Children Diseases, Slovak Medical University, Bratislava, Slovakia

**Keywords:** coronavirus 2019, SARS-CoV2, COVID-19, lymphopenia, eosinopenia, cytokine storm, immunoparalysis

## Abstract

Severe acute respiratory syndrome caused by a novel 2019 coronavirus (SARS-CoV2) represents one of the most studied infectious diseases of today. The number of scientific reports and publications increases exponentially day by day. While the majority of infected subjects are asymptomatic or show mild symptoms, there is an important proportion of patients who requires hospitalization and, sometimes, intensive care. Immune response to novel coronavirus is complex, involves both innate and adaptive immunity, and is biphasic. Significant differences were observed when comparing severe and non-severe patients. Analysis of the reported results from clinical trials clearly show an involvement of specific cellular immunity (predominantly leucopenia, decreased counts of CD3^+^, CD4^+^, and CD8^+^ T lymphocytes, changes of T cell compartment) and the so-called cytokine storm, which is associated with worsening of symptoms and the promotion of lung damage. An interesting finding regarding eosinopenia that can have both diagnostic and prognostic value is reported by some authors. Examination of selected immune parameters could help to identify severe patients with the risk of unfavorable course of the disease, predict the prognosis and recognize improvement in the clinical status. Moreover, detailed analysis of the immune changes could help to select novel prospective therapeutic strategies.

## Introduction – COVID-19 and the Immune System

The world is facing a global pandemic of severe acute respiratory syndrome caused by a novel 2019 coronavirus (SARS-CoV2). The diseases caused by this novel coronavirus was named COVID-19 (Coronavirus Disease 2019). The number of infected subjects increasing exponentially day by day is accompanied by an increasing number of patients in critical status and those who die. It is evident that the immune system plays a crucial role in the response to SARS-CoV2 with significant differences among severe and non-severe patients (Shi et al., 2020). It is suggested that evaluating the selected immune parameters could help us to understand better the dynamics of immune system activation and probably to select the appropriate prognostic markers with respect to disease outcomes and expected complications. Moreover, immune support treatment could be another possible step in the complex management of these patients. On the other hand, it is not clear whether the observed changes in immune parameters are direct consequence of COVID-19 or are predisposing factors for this infection and its severe course. Several questions regarding the immune response and its dynamics remain unresolved and the ongoing studies should bring the answers.

Immune response in SARS-CoV1, MERS and other viral pneumonias similar to SARS-CoV2 were recently reviewed and summarized (Lin et al., 2020; Vardhana and Wolchok, 2020; Yi et al., 2020). However, a complex review of the immune changes in COVID-19 patients with respect to the clinical picture, complications and severity is not yet available. We aimed to summarize the most important changes in inflammatory markers and the selected immune laboratory parameters, which were extracted from the available published literature.

## Immune Response to COVID-19

Immune response to COVID-19 infections consists of two phases: In the beginning, during the phase of incubation and non-severe stages, a specific adaptive immune response is necessary to reach the control over virus proliferation, to eliminate the virus, and to prevent disease progression. Later, in certain patients (with comorbidities, of older age, and probably with specific genetic background) the cytokine release syndrome (so-called cytokine storm) promotes the progression to severe status and organ damage (Shi et al., 2020). Therefore, the host immune system is on one hand essential for the resolution of COVID-19 infection, but on the other can serve as a crucial player in the pathogenesis of the major clinical complications of the disease (Favalli et al., 2020). Several studies have shown various changes in selected immune laboratory parameters during the COVID-19 infection by comparing infected and healthy, asymptomatic and symptomatic, and severe and non-severe patients (Table 1).

**Table 1:** Summary of the most important findings concerning immune parameters in patients with COVID-19.

**Parameters**	**References**
↓ Lymphocytes	Deng et al., 2020; Diao et al., 2020; Ding et al., 2020; Ganji et al., 2020; Giamarellos-Bourboulis et al., 2020; Guan et al., 2020; Han et al., 2020; Huang C. et al., 2020; Huang Y. et al., 2020; Liu K. et al., 2020; Lupia et al., 2020; Mo et al., 2020; Sun S. et al., 2020; Wu and McGoogan, 2020; Yang et al., 2020; Zhang M. Q. et al., 2020
↑ Neutrophils	Mo et al., 2020; Wan et al., 2020
↑ Monocytes	Wen et al., 2020; Zhang M. Q. et al., 2020
↑ CD14^+^ monocytes with inflammatory genes expression and CD14^++^IL-1β^+^ monocytes	Wen et al., 2020
↓ Eosinophils	Liu F. et al., 2020; Li Y. X. et al., 2020; Qian et al., 2020; Qilin et al., 2020; Wang Z. et al., 2020; Yun et al., 2020; Zhang J. J. et al., 2020
↓ CD3^+^ T cells, CD4^+^ T helper, and CD8^+^ T cytotoxic cells	Chen et al., 2020; Giamarellos-Bourboulis et al., 2020; He et al., 2020; Ling et al., 2020; Liu K. et al., 2020; Ouyang et al., 2020; Qin et al., 2020; Wu et al., 2020; Xu et al., 2020; Zeng et al., 2020
↓ memory T helper (CD3^+^CD4^+^CD45RO^+^) and ↑ naïve T helper cells (CD3^+^CD4^+^CD45RA^+^)	Qin et al., 2020
↑ mean fluorescence density of CD8^+^ on T cytotoxic lymphocytes	Ganji et al., 2020
↓ cytotoxic T cells (CD3^+^CD8^+^CD28^+^)	Qin et al., 2020
↑ cytotoxic T cells (CD38^+^HLA-DR^+^CD8^+^)	Zheng M. et al., 2020
↑ production of IFN-γ by CD4^+^ and CD8^+^ T cells in severe and extremely severe cases	Wang F. et al., 2020
↓ production of IFN-γ by CD4^+^ T cells	Chen et al., 2020
↓ T regulatory cells (CD3^+^CD4^+^CD25^+^CD127low^+^)	Gong et al., 2020
↓ natural T regulatory cells (CD45RA^+^) in severe cases	Wang F. et al., 2020
↓ expression of HLA-DR on CD4^+^ T lymphocytes in severe cases	Giamarellos-Bourboulis et al., 2020
↓ B cells	Giamarellos-Bourboulis et al., 2020; He et al., 2020; Wang F. et al., 2020; Xu et al., 2020
↓ NK cells	Giamarellos-Bourboulis et al., 2020; Wang F. et al., 2020
↑ expression of NKG2A on NK cells and CD8^+^ T cells	Qu et al., 2020
↑ PD-1 and TIM-3 on T cells (markers of cell exhaustion)	Diao et al., 2020; Liu et al., 2020b
↑ C3 in severe cases	He et al., 2020
↑ SAA	Xu et al., 2020
↑ IL-10 and IL-6	Diao et al., 2020; Qin et al., 2020; Wang F. et al., 2020
↑ IL-2R, IL-6, IL-8, IL-10 ad TNF-α	Chen et al., 2020; Gong et al., 2020
↑ IL-2, IL-7, IP-10, MCP-1, MIP 1-α, TNF-α	Huang C. et al., 2020
↑ Th17 signature	Wu and Yang, 2020

*IL, interleukin; IP-10, interferon-γ inducible protein 10; MCP-1, monocyte chemoattractant protein 1; MIP-1α, macrophage inflammatory protein 1-α; PD-1, programmed cell death; SAA, serum amyloid A; TIM-3, T-cell immunoglobulin and mucin-domain containing-3; TNF-α, tumor necrosis factor α. ↓, decreased, ↑, increased*.

## Inflammatory Response in COVID-19

COVID-19 activates the immune system via different receptors; among them, Toll-like receptors (TLR-3, 4, and 7) are some of the most crucial. The binding of COVID-19 to TLRs activates the formation of active IL-1β and IL-6. These two cytokines are the central proinflammatory molecules causing the systemic clinical symptoms (malaise, fever, myalgia, etc.) and also leading to inflammation of the lungs (Conti et al., 2020).

Increased C-reactive protein (CRP) and high-sensitivity CRP is reported in the majority of COVID-19 patients. The highest values are usually observed in the most severe cases. The elevation of other inflammatory cytokines and chemokines, IL-2R, IL-6, IL-8, IL-10, and TNF-α, was found especially in severe cases compared to mildly affected individuals (Chen et al., 2020; Gong et al., 2020). Severe COVID-19 cases yielded higher concentrations of inflammatory markers compared to moderate patients (Chen et al., 2020). A cytokine profile similar to that of patients with secondary hemophagocytic lymphohistiocytosis, including increased IL-2, IL-7, interferon-γ-inducible protein 10, monocyte chemoattractant protein 1, macrophage inflammatory protein 1-α, and TNF-α was reported by Huang C. et al. (2020). Among non-survivors, an elevation of serum ferritin and IL-6 was one of the significant predictors (Ruan et al., 2020). Therefore, it could be suggested that, besides antiviral treatment, a balanced immunosuppression (e.g., by using the inhibitors of selected cytokines – IL-6, IL-1) could be another effective approach to reduce and modulate viral induced hyperinflammation (storm) and thus to prevent severe and irreversible organ damage that contributes to the relatively high mortality of COVID-19 patients (Conti et al., 2020; Mehta et al., 2020). Moreover, based on the previous experience with SARS, the cytokine storm is strongly associated with pulmonary inflammation and excessive lung damage. Similar to MERS-CoV and SARS-CoV-1, inflammation in COVID-19 patients is also characterized by Th17 cytokine signature. Therefore, a selective JAK inhibitor (e.g., fedratinib) could present an option for treatment of these patients with severe course by affecting the production of Th17-cytokines (Wu et al., 2020). On the other hand, several authors showed that the early stage of inflammatory response to SARS-CoV-2 is characterized by weakened interferon production from the infected cells which can result in the progression of the infection (Chu et al., 2020). In contrast with the other pro-inflammatory cytokines that should be therapeutically decreased and blocked, application of interferons (e.g., interferon lambda) could present another therapeutic strategy, especially in the early stages of COVID-19 (O'Brien et al., 2020).

## White Blood Cell Counts and COVID-19

Substantial numbers of clinical studies and observations have reported lymphopenia in a significant proportion of patients with confirmed COVID-19 infection (Chen et al., 2020; Ding et al., 2020; Guan et al., 2020; Han et al., 2020; Huang C. et al., 2020; Lin et al., 2020; Liu K. et al., 2020; Lupia et al., 2020; Mo et al., 2020; Sun S. et al., 2020; Wu and McGoogan, 2020; Yang et al., 2020; Zhang M. Q. et al., 2020). Lymphopenia could be considered as a signature for severe COVID-19 infection and pneumonia (Bermejo-Martin et al., 2020). The highest proportion of lymphopenic patients (83.2%) was reported by Guan et al. (2020). In a group of 10 patients with COVID-19 pneumonia, the authors investigated the differential white blood cells counts. COVID-19 patients had lower absolute numbers of lymphocytes and eosinophils compared to the other 30 patients with non-COVID-19 pneumonia (Li Y. X. et al., 2020). Comparing mild and severe cases of COVID-19, a more profound decline in the absolute number of lymphocytes was particularly observed in severe cases and critically ill patients (Chen et al., 2020; Peng et al., 2020; Wan et al., 2020). In contrast, severe cases were frequently characterized by neutrophilia (Mo et al., 2020; Wan et al., 2020). Monocytosis was also observed sporadically in this group of patients (Zhang M. Q. et al., 2020). During the early recovery stage of COVID-19, an increase of CD14^+^ monocytes with inflammatory gene expression as well as abundance of CD14^++^IL-1β^+^ monocytes were recently described (Wen et al., 2020). Patients with abnormal lung imaging findings had lower lymphocytes compared to patients without lung involvement (Zhang X. et al., 2020). Lymphopenia was also seen in a small group of COVID-19 patients co-infected with influenza. Interestingly, the course of the disease and laboratory and imaging findings were similar to those with COVID-19 only (Ding et al., 2020). The proportion of lymphocytes was significantly lower in the elderly group compared to the young and middle-aged groups (Liu K. et al., 2020). Compared to adult patients, infants and young children usually have a mild course of disease without reductions in leukocyte or lymphocyte counts (Zhou et al., 2020a,b). However, up to now, only a few reports containing detailed data about children infected with this novel virus are available in the literature, so conclusions cannot be drawn. It is evident, that in children the immune response to COVID-19 is different compared to the adults and majority of the children have mild symptoms or asymptomatic course. This could be explained by higher capacity to produce natural antibodies with broad reactivity, presence of physiologic lymphocytosis and differences in lymphocyte compartment, virus-to-virus interactions in the airways (simultaneous presence of other respiratory viruses in the airway mucosa), differences in the expression of *ACE2*, different inflammatory response in children and many others aspects (Brodin, 2020; Carsetti et al., 2020). On the other hand, recently described COVID-19-associated pediatric multi-system inflammatory syndrome shows several differences to the course of COVID-19 in adults and is highly specific for children (Deza Leon et al., 2020).

Another interesting finding reported by several author groups is the eosinopenia associated with COVID-19 infections (Jesenak et al., 2020). Recent findings suggest that eosinophils have important antiviral properties (Flores-Torres et al., 2019). Some eosinophil-derived granular proteins (e.g., eosinophil-derived neurotoxin, eosinophil cationic protein) show antiviral activity against single-stranded RNA viruses. Moreover, eosinophils are able to produce nitric oxide and can induce CD8^+^ T cell proliferation and activation as a response to virus- or viral-peptide exposure. Eosinophils can also support viral clearance (Jesenak and Schwarze, 2019). Taking into account all these facts, the reported eosinopenia in COVID-19 patients is of special interest (Du et al., 2020; Li Y. X. et al., 2020; Qian et al., 2020; Zhang J. J. et al., 2020). Li Y. X. et al. (2020) compared 10 COVID-19 patients with 30 patients affected by other viral pneumonia. They found that leukopenia, lymphocytopenia, and eosinopenia were more common in COVID-19 patients compared to non-COVID-19 subjects. Detailed investigations of 140 hospitalized patients suggested that eosinopenia with lymphopenia may be a potential indicator for COVID-19 with both diagnostic and prognostic value (Zhang J. J. et al., 2020). Combination of eosinopenia together with elevated high-sensitivity CRP could effectively triage suspected patients with COVID-19 from the other patients with fever (Qilin et al., 2020). Eosinopenia indicated poor prognosis also in another study (Du et al., 2020). The increase in eosinophils observed during treatment from the initially low levels could be a positive indicator of clinical improvement (Liu F. et al., 2020; Sun S. et al., 2020). On the other hand, pooled analysis suggested that eosinopenia may not be associated with unfavorable progression of COVID-19 (Lippi and Henry, 2020). The role of eosinophils in COVID-19 is still discussed. It seems that observed eosinopenia has more diagnostic and eventually prognostic value then real participation on the COVID-19 pathology (Lindsley et al., 2020; Qian et al., 2020). Whether it is the consequence of the block in eosinopoiesis, result of the decreased release of eosinophils from bone marrow or IFN-induced apoptosis remains to be answered in further studies.

## Specific Cellular Immunity Parameters

Based on the current knowledge that T lymphocytes play a central role in the protection against coronaviruses, it is of special interest to analyze the possible relationship of lymphocytes and their subpopulations relative to the clinical course and complications of COVID-19. T cells are mostly affected by COVID-19 (Chen et al., 2020; Ouyang et al., 2020; Qin et al., 2020; Sun D. W. et al., 2020; Sun S. et al., 2020; Wu and McGoogan, 2020). A study in 752 patients analyzed the subpopulations of lymphocytes (CD3^+^, CD4^+^, CD8^+^). They compared the findings in COVID-19 patients with the normal reference values of the Chinese population. CD3^+^ lymphocytes below 900 cells/mm^3^, CD4^+^ cells below 500 cells/mm^3^, and CD8^+^ lymphocytes below 300 cells/mm^3^ were considered to select the subjects at higher risk of COVID-19 infection. Significant differences in the numbers of CD4^+^ and CD8^+^ T lymphocytes between mild and severe/critical COVID-19 cases were described (Chen et al., 2020). No differences between B-cells and NK cell numbers were usually found (Sun D. W. et al., 2020; Sun S. et al., 2020; Zeng et al., 2020), however, recent studies reported also the decline in B cells numbers (Xu et al., 2020). Another authors' group reported that the non-intensive care unit patients with total T cells, CD4^+^ and CD8^+^ cell counts lower than 800, 400, and 300/μL, respectively, require attention and intervention even in immediate absence of more severe symptoms due to a high risk for further deterioration (Diao et al., 2020). Moreover, lower counts of T lymphocytes and their subsets (total CD3^+^ <200/μL, CD4^+^ <100/μL, and CD8^+^ <100/μL) were significantly associated with higher risk of in-hospital death due to COVID-19. The warning values to predict in-hospital death of lymphocytes, CD3^+^, CD4^+^, CD8^+^, and CD19^+^ cells were 559, 235, 104, 85, and 82, respectively (Xu et al., 2020). Similar results found also He et al. (2020). In several studies, a decline in CD8^+^ was more frequently observed in COVID-19 patients than a decline in CD4^+^. Decreased numbers of CD8^+^ T lymphocytes were also reported by other groups, especially in connection with the presence of lung involvement and pneumonia development (Liu et al., 2020a). CD8^+^ T cytotoxic cells can contribute to the elimination of virus by production of many biologically active molecules, such as perforins, granzymes and interferons. Therefore, the decline number of CD8^+^ T cells along with the dysfunction could significantly contribute to the severe course of COVID-19 and loss of control over virus production. An interesting observation brought a study of Ganji et al. (2020). While no differences in CD4:CD8 ratio, CD4^+^ and CD8^+^ T cell numbers and CD4 mean fluorescence intensity did not differ between COVID-19 patients and healthy individuals, mean fluorescence intensity of CD8 expression on T cytotoxic cells increased significantly in COVID-19 infected patients. This could the sign of hyperactivation of T cytotoxic cells as a response to SARS-CoV2 infection (Ganji et al., 2020). In contrast, a series of other studies found more significant differences in CD4^+^ T helper cell numbers (Qin et al., 2020). Moreover, decreased production of IFN-γ by CD4^+^ T cells were observed in the severe cases (Chen et al., 2020). A detailed analysis of T cell compartment showed the analysis of the laboratory results of 452 COVID-19 patients, among which 286 were diagnosed with severe infection. Severe cases had significantly decreased T cells, especially CD4^+^ T helper cells. The percentage of naïve helper T cells (CD3^+^CD4^+^CD45RA^+^) increased, and memory helper T cells (CD3^+^CD4^+^CD45RO^+^) decreased in severe cases. Similarly, CD28^+^-positive cytotoxic T cells (CD3^+^CD8^+^CD28^+^) were decreased in severe cases. Moreover, COVID-19 patients had lower level of regulatory T cells (CD3^+^CD4^+^CD25^+^CD127low^+^), especially in severe cases. No differences were found for activated helper or suppressor T cells (expressing HLA-DR^+^). Interestingly, compared to the changes in absolute numbers of T cells and NK cells, their functions (indicated by PMA/Ionomycin-stimulated IFN-γ cells) remained within the normal range, with no differences between severe and non-severe cases (Qin et al., 2020). The percentage of natural T regulatory cells (CD45RA^+^ Treg) was significantly decreased in severe patients, especially in those with extremely severe illness (Wang F. et al., 2020). Regulatory T cells are crucial in the maintenance of the immune homeostasis in various T cells subpopulation and their activity and they determine the effective and balanced immune response. Therefore, the decreased number and function of T regulatory cells are of special interest as a potential therapeutic target. The same results regarding the decline in T cells and CD4^+^ T helper cells was also reported in the study by Wu et al. (2020). It was suggested that the absolute number of CD4^+^ T lymphocytes may predict the duration of viral RNA in the stools of infected patients: The lower the CD4^+^ count, the longer the duration of viral RNA persistence in the stool (Ling et al., 2020). A pathological autopsy report of three COVID-19 patients who died due to pneumonia showed that the alveoli were infiltrated mainly with macrophages and monocytes with minimal infiltrating lymphocytes, eosinophils and neutrophils. Most of the infiltrated lymphocytes were CD4^+^ T cells (Yao et al., 2020). Another important immunopathology that could contribute to the immune response to COVID-19 is the Th17 immune response activated in the environment of overproduction (Hotez et al., 2020). The response of immune system and susceptibility to different infections is determined by many factors, among them HLA haplotypes play an essential role. It could be suggested that the specific cellular response would be modified by specific HLA haplotypes, which can affect the characteristics of the developing anti-viral immunity and its efficacy in the achievement of control over viral infections (Shi et al., 2020). T-cell activation is probably compromised by infected antigen presenting cells (Shin et al., 2019). The observed lymphopenia could have various explanations. It could be the results of virus induced apoptosis or so-called pyroptosis (e.g., cell death induced by IL-1β). Moreover, also the initial viral load could probably modify the final immune response.

Only a few studies have also analyzed the functional changes of lymphocytes. As referred to above, in a study of Qin et al. (2020), despite significantly decreased lymphocyte counts, their function was not diminished. The kinetics of immune response in relation to clinical and virological features in one patient with mild-to-moderate COVID-19 was recently published in detail (Thevarajan et al., 2020). The authors described an increase in antibody-secreting cells, follicular helper T cells, activated CD4^+^ and CD8^+^ T cells, and immunoglobulin IgG- and IgM-binding COVID-19 in the blood before symptomatic recovery. A rapid increase in the co-expression of HLA-DR and CD38 on CD8^+^ T cells (CD38^+^HLA-DR^+^CD8^+^) was detected before clinical status improvement. This phenotype of cells produced large amounts of potent antimicrobial agents (granzymes A and B, perforin; Thevarajan et al., 2020). In severe patients with respiratory failure, expression of HLA-DR on CD4^+^ T cells was markedly decreased. Moreover, SARS-CoV-2 patient plasma inhibited HLA-DR expression (Giamarellos-Bourboulis et al., 2020). All the cited studies are confirming that the associated cytokine storm negatively affects the activation and effective functions of the immune cells. A selective inhibition of particular cytokine could have profound effect on the restoring the immune functions and improving the clinical status.

Other possible consequence of insufficient immune control over SARS-CoV-2 infection could be also the anergy and exhaustion of immune cells, which can be measured by various surface markers. NKG2A (C-type lectin receptor with inhibitory effects) expression on NK cells and CD8^+^ cells affects their activation. In COVID-19 patients, increased expression of NKG2A on NK cells and CD8^+^ led to functional exhaustion of both cell populations. During the convalescent period, the total numbers of NK cells and CD8^+^ T lymphocytes increased, accompanied by decreased expression of NKG2A. These findings suggest that the downregulation of NKG2A expression may correlate with disease control and improvement in clinical status (Zhang X. et al., 2020). Whether this is a direct effect of COVID-19 remains unclear; however, it is clear that protective antiviral immunity is strongly affected in these patients. This could be the result of the direct effect of coronavirus, or it could be the result of the associated cytokine storm. Targeting NKG2A may prevent the functional exhaustion of antiviral immune cellular protection and contribute to virus elimination. Other study showed that besides dramatically decreased numbers of total T cells, CD4^+^ and CD8^+^ T cell subsets, T cells from COVID-19 patients had significantly higher levels of the exhausted marker PD-1 (programmed cell death 1; CD279) compared to healthy controls. Moreover, gradual increase of PD-1 and another co-inhibitory receptor TIM-3 (T-cell immunoglobulin and mucin-domain containing-3; CD366) expression on the T cells was accompanying the progression from the prodromal to severe symptomatic stages (Diao et al., 2020). This suggests that the exhaustion of T cells (so-called immunoparalysis) could contribute to the ineffective immune control over virus replication and to the progression of the diseases into severe stages. Therefore, therapeutic strategies blocking PD-1/PD-1L and TIM3 pathways could have a significant impact on the outcome of COVID-19 patients with low-to-medium expression of these markers. On the other hand, the patients with high expression of CD366 (TIM-3) would have worse prognosis with limited effect of immune interventions (Chiappelli et al., 2020). Similar results published also another group (Liu et al., 2020a). Besides the measurement of the surface markers on the cells, interesting results brought the study evaluating the expression of different genes during the COVID-19 progression and subsequent treatment. During the disease progression, a down-regulation of the genes involved in Th17 cell differentiation, cytokine-mediated signaling and T cell activation was evident. After the treatment in severe cases, *MAP2K7* and *SOS1* (both involved in the T-cell activation and signaling) were upregulated (Ouyang et al., 2020).

## Immunoglobulins and Complement

Surprisingly, immunoglobulin values and complement components are only occasionally reported in the published cohorts, probably due to normal observed levels. Qin et al. (2020) did not see any changes of immunoglobulin isotypes (IgG, IgA, IgM) and complement proteins (C3, C4) in COVID-19 patients. No differences were found when comparing mild and severe patients, with the exception of IgM, which was lower in severe cases (Qin et al., 2020). A certain time before clinical improvement, and increases in antibody-producing cells and specific immunoglobulins (IgM, IgG) binding COVID-19 could be observed (Thevarajan et al., 2020). In severe cases, increased level of C3 can probably represents a possible regulatory factor in the context of systemic inflammation (He et al., 2020).

## Immune Parameters as Prognostic Factors

In a study by Qu et al. (2020), positive correlations between platelet-to-lymphocyte ratio (PLR), peak platelet numbers and severity of the disease were confirmed. The average hospitalization days of patients with platelet peaks were longer than those without; moreover, the patients with platelet peaks were older compared to those without. It was confirmed that the greater the difference between the PLR at admission and during treatment, the greater possibility of severe pneumonia. The authors suggested that the platelet peaks could be related to the cytokine storm described in COVID-19 infection (Qu et al., 2020). The early clinical and laboratory findings of COVID-19 pneumonia are low-to-midgrade fever, dry cough, and fatigue, with normal white blood cell count, reduced lymphocyte count and elevated CRP (Han et al., 2020). Eosinopenia could represent another prognostic factor (Du et al., 2020) and the increase of eosinophils may serve as a positive predictive factor of clinical improvement (Liu F. et al., 2020; Sun S. et al., 2020). Detailed analysis of the immune parameters' changes identified certain possible prognostic factors. Several authors identify a degree of T-cell decline to be a negative predictive factor for disease course (Diao et al., 2020; He et al., 2020; Xu et al., 2020). The changes of the expression of inhibiting markers (e.g., NKG2A, TIM-3) on the cell surface could serve as another possible prognostic factor (Chiappelli et al., 2020; Liu et al., 2020a; Zheng M. et al., 2020). It should be concluded, that early recognition of the immune phenotype associated with disease progression could help to identify the most severe and risky patients, with the modification of the treatment procedure.

## Immuno-Interventional Approach in COVID-19 Treatment

It is evident that the treatment approach should consist of the combination of several drugs potentially affecting the particular components of COVID-19 infections (Table 2). The majority of the protocols include the application of **antiviral drugs** (mainly oseltamivir, ganciclovir, lipinavir/ritonavir, umifenovir, fevipiravir, and experimental drug remdesivir), **anti-malarial medicaments** (chloroquine phosphate, hydrochloroquine; Cunningham et al., 2020) or **azalides** (azithromycin). Besides these approaches, **cytokine-targeted monoclonal antibodies** (anti-IL-1 – anakinra, cakakinumab or anti-IL-6 – tocilizumab) and **JAK inhibitors** (e.g., fedrotinib) are also considered as potential therapies (Conti et al., 2020; Thevarajan et al., 2020; Wu et al., 2020). These treatments are especially for moderate and severe cases requiring hospitalization.

**Table 2:** Current possibilities and approaches in the treatment^*^ of COVID-19 (adapted and modified from Cunningham et al., 2020; Jayawardena et al., 2020; Ye et al., 2020).

Anti-viral agents	Lopinavir/ritonavir; umifenovir; fevipiravir; oseltamivir; ganciclovir; remdesivir
Anti-malarials	Chloroquine phosphate, hydrochloroquine
Azalides	Azithromycin
Targeted therapies	•Monoclonal antibodies against IL-1 (anakinra, canakinumab) •Monoclonal antibodies against IL-6 (tocilizumab) •TNF-α blockers •JAK inhibitors (e.g., fedratinib)
Others possibilities	•Intravenous immunoglobulins •Interferons and their inhibitors •Convalescent donor plasma
Natural and synthetic immunomodulators	•Minerals (zinc, selenium) •Biologically active polysaccharides (β-glucans) •Vitamins (vitamin D, vitamin C, vitamin A) •Phytopharmacs (*Pellargonium sidoides* and others) •Synthetic immunomodulators (inosine pranobex) •Traditional Chinese medicine

* *The majority of the reported medicaments or treatments are used experimentally and off-label in the indication of COVID-19, since randomized placebo-controlled trials are missing*.

However, are there any possibilities that may support the immune system in mild cases? Despite the lack of clinical data, certain studies and experimental data suggested the potential role of **zinc** in coronavirus infections. Recent review summarized the possible useful mode of actions of zinc in the management of COVID-19 (Skalny et al., 2020). Zinc inhibits RNA polymerase activity and blocks the virus replication *in vitro* (Te Velthuis et al., 2010). Zinc ions also show an ability to inhibit SARS-COV protease (Lee et al., 2007). Moreover, the general support of anti-viral immunity (e.g., production of interferons) and complex anti-inflammatory activity by inhibiting NF-κB signaling could be beneficial in the context of known effects of SARS-CoV2 in the organisms (Skalny et al., 2020). Zinc could improve the efficacy of chloroquine and hydroxychloroquine which are currently used in COVID-19 infection (Shitti and Afolami, 2020).

**Biologically active polysaccharides** (e.g., **β-glucans**) represent a highly studied group of natural immunomodulators with pluripotent biological activities. Certain molecules are able to attenuate inflammatory cytokine release and prevent lung injury in animal models (Bedirli et al., 2007; Cao et al., 2018) and restore the cytokine imbalance by promoting the secretion of anti-inflammatory compounds (Chen et al., 2013). Another mode of action could be the support of NK cell functions and modulatory effect on T cells (Bobovak et al., 2010; Bergendiova et al., 2011; Jesenak et al., 2013). A recent study confirmed the role of pleuran (β-glucan isolated from *Pleurotus ostreatus*) in the treatment of acute herpes simplex type 1 infection (Urbancikova et al., 2020). Using *in vitro* model of lung injury, lentinan (β-glucan from *Lentinus edodes*) reduced cytokine-induced NF-κB activation in human alveolar epithelial cells and attenuated pro-inflammatory cytokine production (TNF-α, IL-2, 6, 8, 22) as well as TGF-β and IL-10 (Murphy et al., 2020). A possible role of α-glucans has also been suggested recently (Di Pierro et al., 2020).

A special interest is focused on the possible role of **vitamin D** and its deficiency in the risk for COVID-19 and its severe course. Vitamin D has pluripotent modulatory activities on both innate and specific immunity. Its deficiency is a risk factor for exaggerated and persistent inflammation (Grant et al., 2020). Moreover, its deficiency increases the risk of severe course of COVID-19 and could at least partly explain the geographic variations in the case fatality rate of COVID-19 (Garg et al., 2020; Marik et al., 2020). Taking into consideration all of these facts, supplementation of vitamin D in an appropriate dose could have both preventive and therapeutic effect in COVID-19 (Ilie et al., 2020; Silberstein, 2020). Another vitamin with potential role in the management of COVID-19 could be **vitamin C**. It was shown that vitamin C is beneficial to critical care management (Nabzdyk and Bittner, 2018) and shortened the intensive care unit stay with the reduction in the mortality rate (Marik et al., 2017). Vitamin C reduced the mortality rate in the patients with sepsis-related ARDS (Kim et al., 2018). Therefore, several authors suggested the potential role of high-dose vitamin C in the treatment and prevention of COVID-19 along with the ongoing clinical trials (Carr, 2020).

Certain **phytotherapeuticals** (e.g., *Pelargonium sidoides* extract) have also yielded a positive effect on the proliferation of coronaviruses (Michaelis et al., 2011). Several authors have also suggested the potential role of selected **Chinese herbal medicines** (Li R. et al., 2020; Zhang D. H. et al., 2020).

**Synthetic immunomodulators** directed at a specific cellular immunity could represent another option. Some of them (e.g., inosine pranobex) showed activity against a broad spectrum of respiratory viruses (Beran et al., 2016).

## Conclusions and Perspectives

The novel coronavirus mainly acts on lymphocytes, especially T cells. Analysis of lymphocyte subsets could be helpful in the early screening of the potential critical course of COVID-19 disease. The consumption of CD4^+^ and CD8^+^ cells, increased concentration of a broad spectrum of proinflammatory cytokines and chemokines, and decreased T regulatory cells could contribute to the excessive inflammatory response (cytokine storm, cytokine release syndrome) with a loss of control over the damaging immune response and the promotion of tissue damage (e.g., in the lungs). Besides other reported laboratory anomalies associated with the unfavorable progression of COVID-19 (e.g., decreased albumin, increased lactate dehydrogenase, alanine aminotransferase, aspartate aminotransferase, bilirubin, creatinine, cardiac troponin, D-dimer, procalcitonin, and CRP; Lippi and Plebani, 2020), special attention has to be paid to the parameters of the immune system. Decreased lymphocyte and eosinophil counts, with deterioration of other parameters of cellular immunity, should be carefully assessed and regularly checked in COVID-19 patients to monitor the course of the disease and predict worsening of the symptoms.

## Author Contributions

MJ was involved analysis and interpretation of the data, literature search, and in drafting and revising the manuscript. MB was involved in analysis and interpretation of the data discussed in the manuscript, and in revising the manuscript. IU was involved in the literature search and in drafting and revising the manuscript. ZR was involved in the literature search, critical analysis of the data, and in revising the manuscript. JV was involved in revising the manuscript. AB was involved in drafting and revising the manuscript. RO was involved in analysis and interpretation of the data and in revising the manuscript. PB was involved in analysis of the data and in revising the manuscript. All the authors have approved the final version of the manuscript.

## Conflict of Interest

ZR was employed by Pneumo-Alergo Centre Ltd. The remaining authors declare that the research was conducted in the absence of any commercial or financial relationships that could be construed as a potential conflict of interest.
